# Supplementary material for: Photokinetics of Photothermal Reactions
Source: Molecules. 2025 Jan 15;30(2):330. doi: 10.3390/molecules30020330 (PMC11767552; doi:10.3390/molecules30020330)
Supplement: Supplementary file 1 [file molecules-30-00330-s001.zip › molecules-3350038-supplementary.pdf]

Photokinetics of photothermal reactions

Mounir MAAFI

Leicester School of Pharmacy, De Montfort University, The Gateway, Leicester, LE1 9BH and UK;
mmaafi@dmu.ac.uk

Examples of reaction photokinetics and elucidation

SI: S1

Photokinetics of $XY_2(4\Phi, 2k)$ reaction

Reaction:

$XY_2(4\Phi, 2k)$

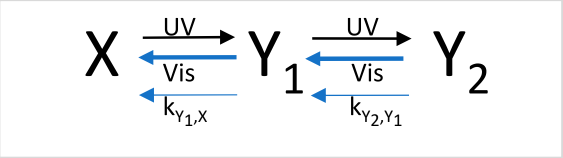

reaction parameters:

$C_X^{Lp,\Delta\lambda,T}(0)$ / M	l_{irr} / cm	$k^T_{Y_1 \to X}$ / s^{-1}	$k^T_{Y_2 \to Y_1}$ / s^{-1}	h / s	λ_{obs} /nm	l_{obs} / cm
1.46 10^{-5}	1.71	0.015	0.007	0.3	344	1.71
$P_{0,tot}$	6.77 10^{-5}					

Fitting equation:

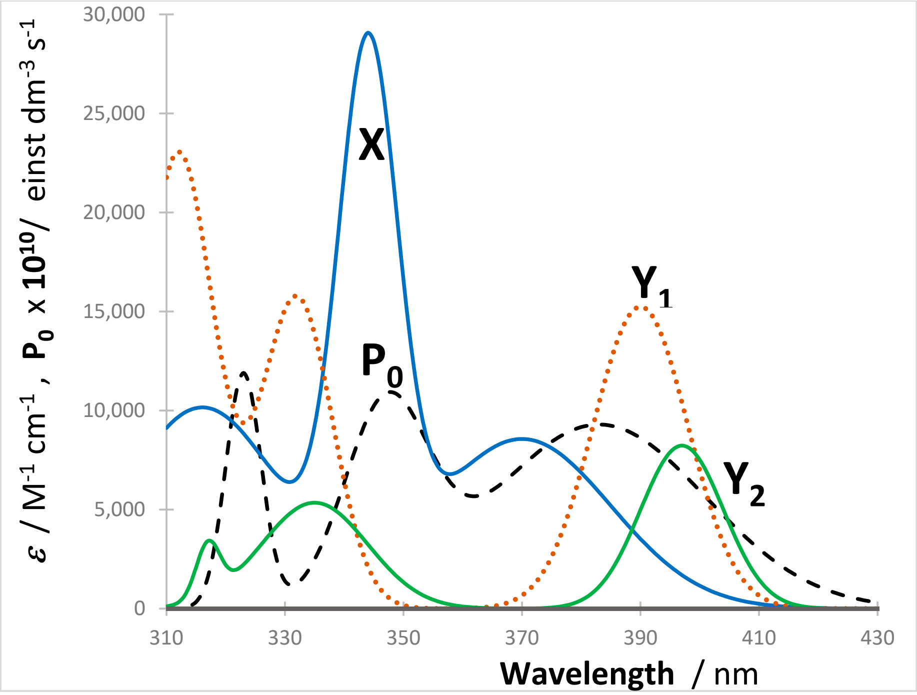

Fig. SI-S1-1: reaction parameters:

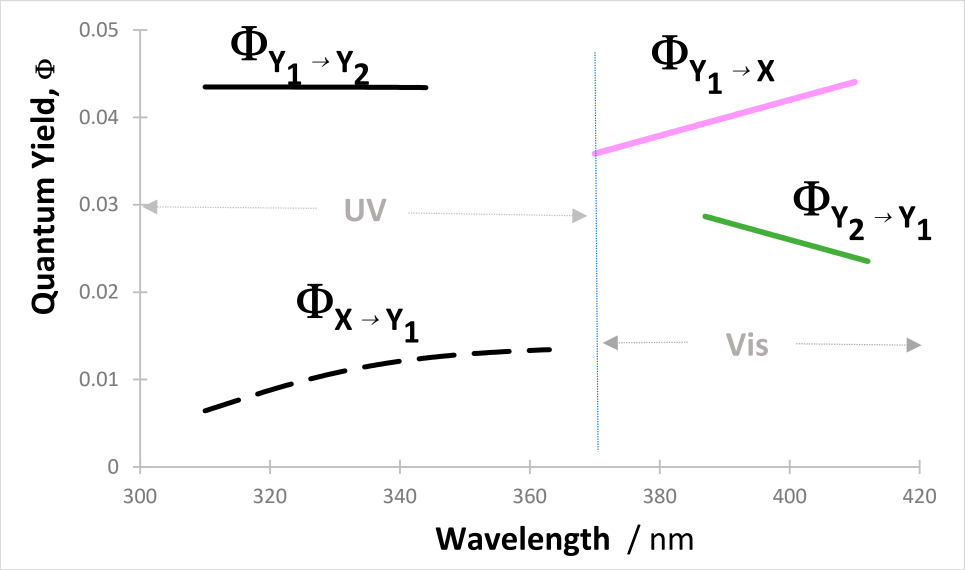

Fig. SI-S1-2:

Fitting equation:

$$C(t) = ww + w_1 \log(1 + cc\, e^{-k_1 t}) + w_2 \log(1 + cc\, e^{-k_2 t}) + w_3 \log(1 + cc\, e^{-k_3 t}) + s_1\, e^{-k_{s1} t} + s_2\, e^{-k_{s2} t}$$

Po = **6.77E-05**

	cc	k_1	k_2	k_3	w_1	w_2	w_3	ww	k_{s1}	k_{s2}	s_1	s_2
**CX**	0.0407	0.0758	0.0116	0.0116	1.53 E-04	0.0112	-0.012	8.35 E-06	0.0123	0.0678	1.89 E-05	-9.92 E-07
**CY1**	0.9555	1.106	1.586	0.8219	-1.90 E-06	-1.251 E-04	-0.00026	2.73 E-06	0.8484	0.05703	0.00011	-2.34 E-06
**CY2**	1.883	0.02716	0.5144	0.5144	1.53 E-07	0.1704	-0.1704	3.53 E-06	0.01347	0.0746	-4.36 E-06	7.54 E-07
**Atot**	0.3484	0.059	0.0966	0.00867	0.194	0.432	0.00091	0.444	0.0135	0	0.199	0

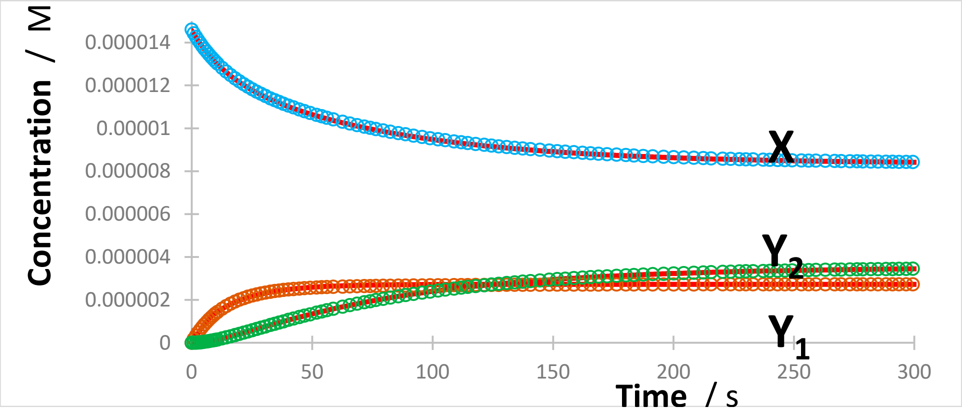

Fig. SI-S1-3:

Co effect:

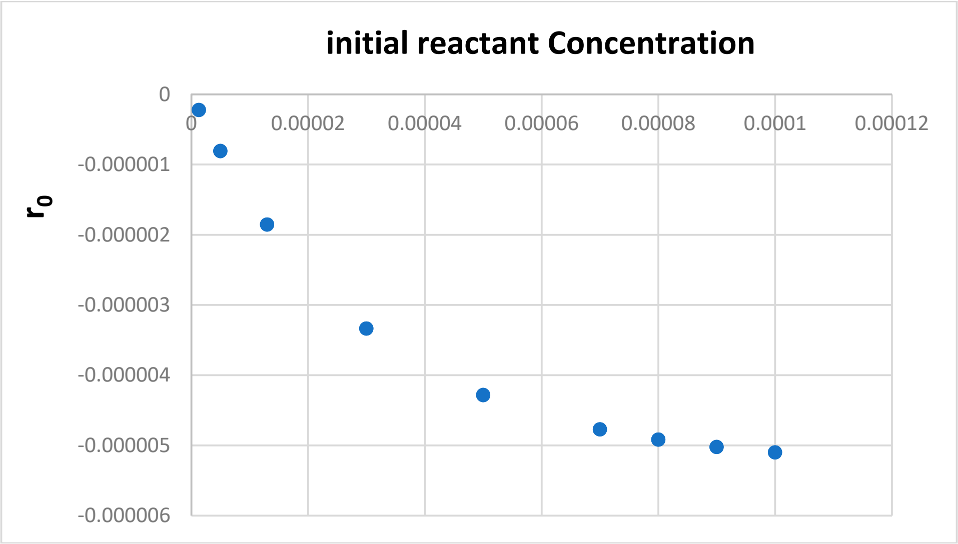

Fig. SI-S1-4:

Po effect: on concentration traces

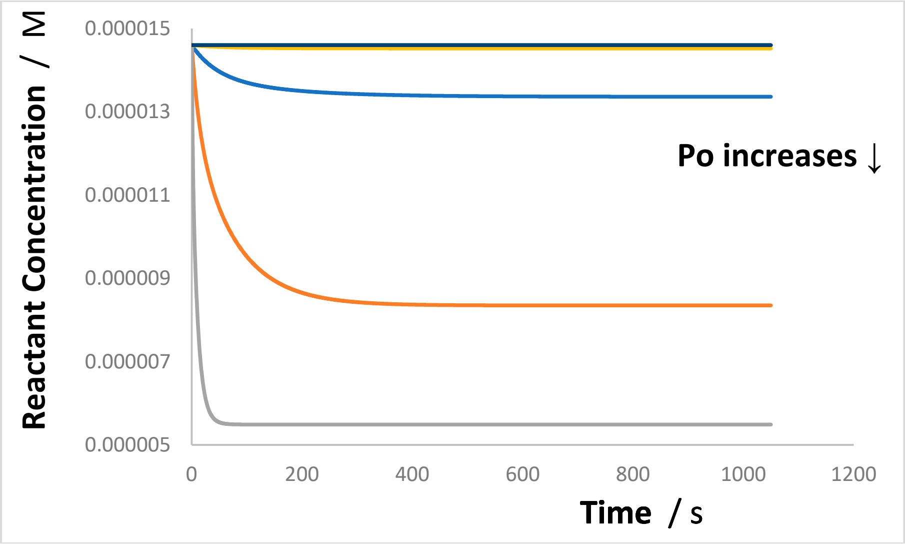

Fig. SI-S1-5:

Po effect:

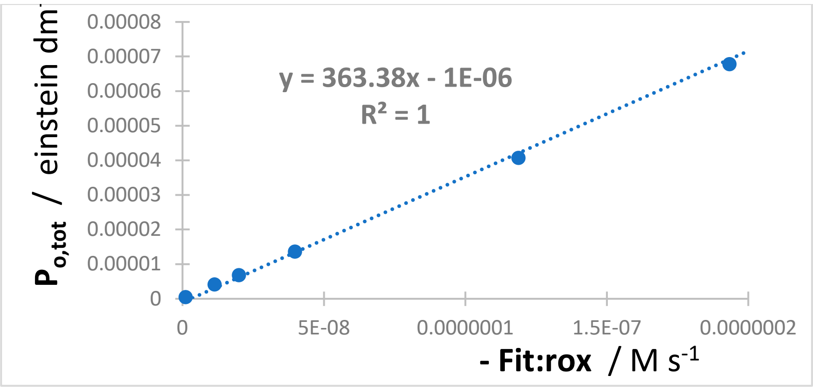

Fig. SI-S1-6:

Po effect: for different reactions

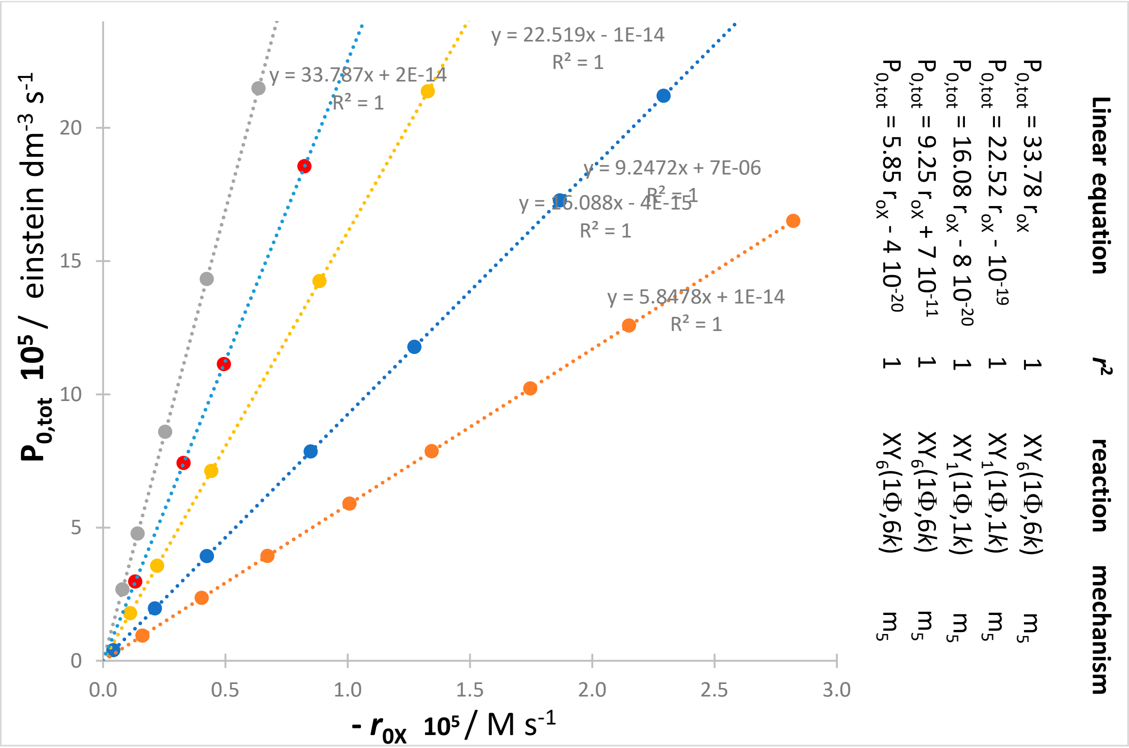

Fig. SI-S1-7:

Po effect: on the Initial rate

roA were multiplied by a factor: x 10^{-4}

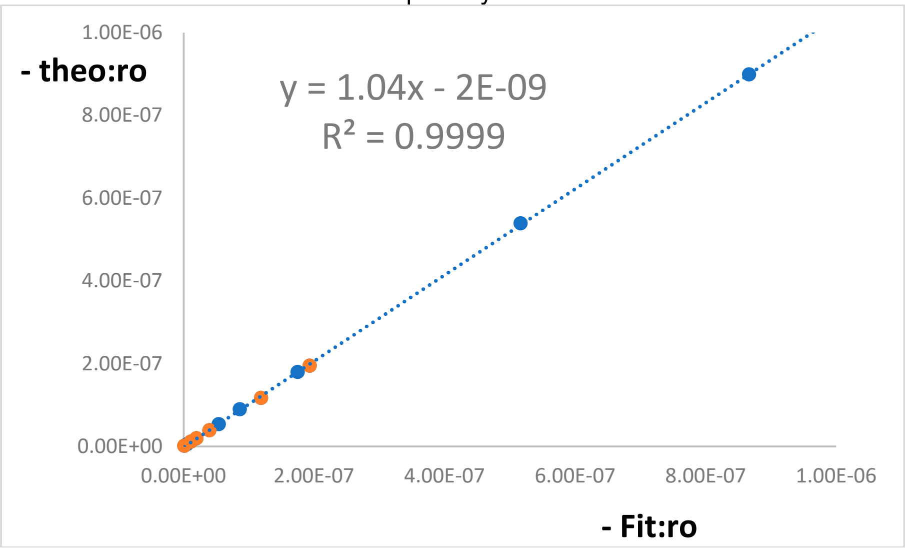

Fig. SI-S1-8:

PY:

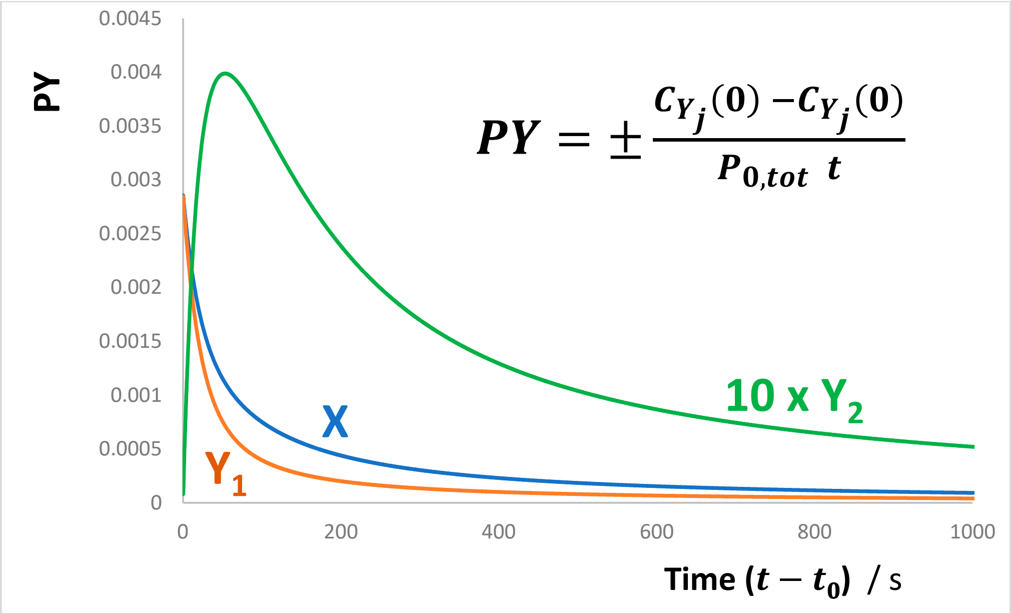

Fig. SI-S1-9:

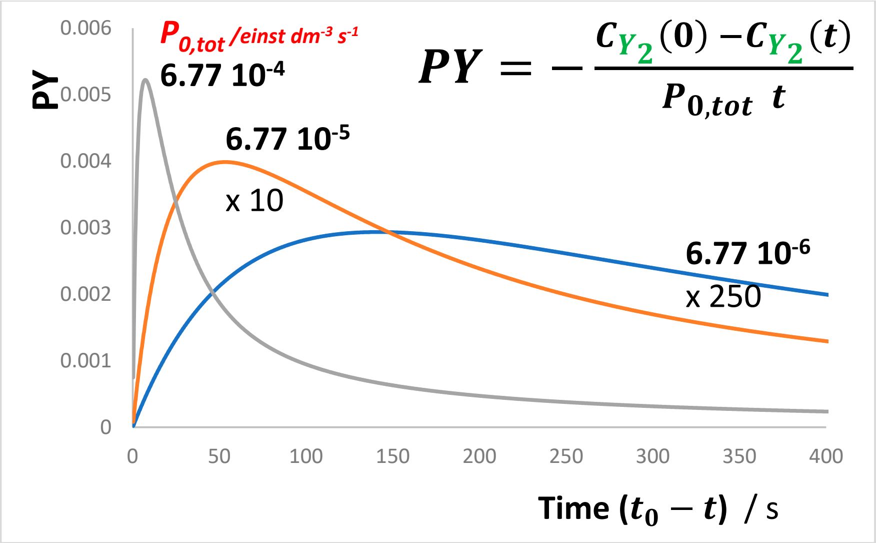

Fig. SI-S1-10:

Effect of Temp.:

roX	-1.95E-07	-1.95E-07	-1.95E-07	-1.95E-07	-1.95E-07	-1.95E-07
CooX	8.34938E-06	1.014E-05	1.185E-05	9.375E-06	1.103E-05	1.142E-05
k(thermal) Y1X	0.015	0.03	0.06	0.0225	0.045	0.0525
k(thermal) Y2Y1	0.007	0.014	0.028	0.0105	0.0175	0.0204167

Effect of Temp.: on concentration traces

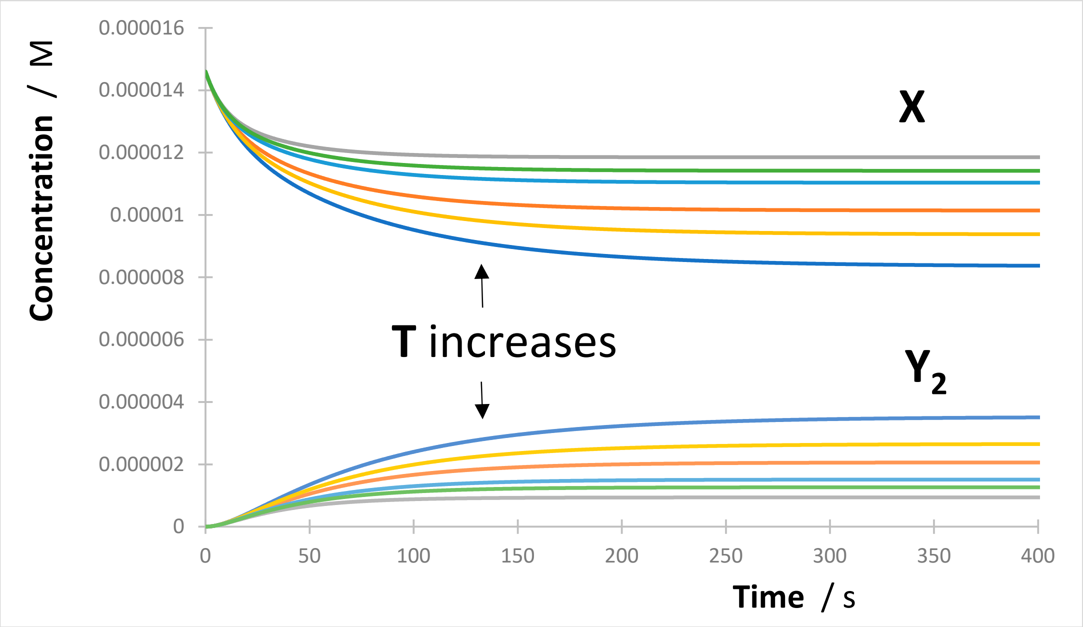

Fig. SI-S1-11:

effect of T: on PY (concentration data)

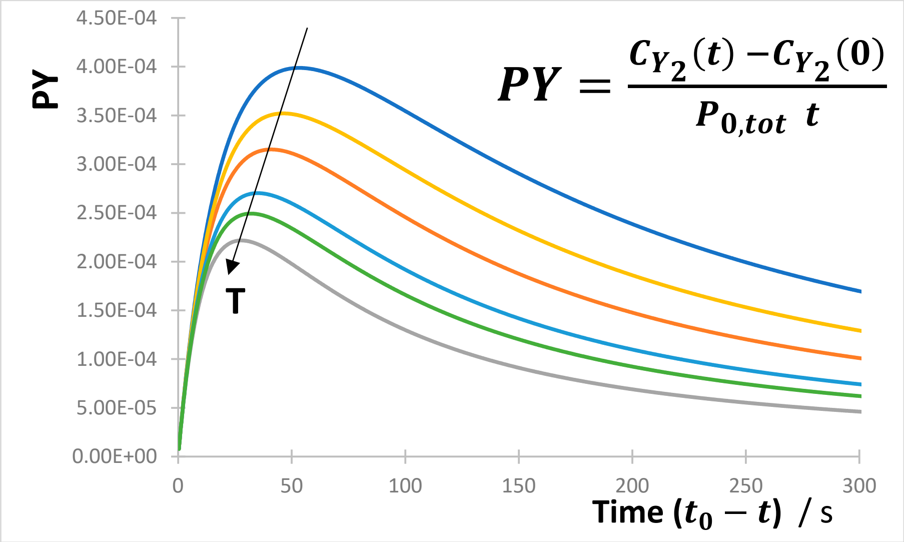

Fig. SI-S1-12:

effect of T: on PY (absorption data)

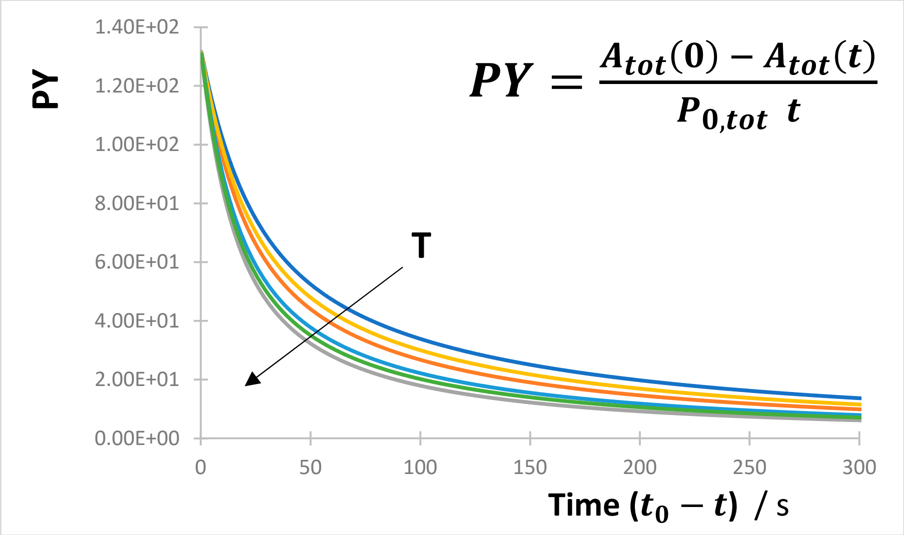

PY
1.40E+02
1.20E+02
1.00E+02
8.00E+01
6.00E+01
4.00E+01
2.00E+01
0.00E+00
0
50
100
150
200
250
300
T
$PY = \frac{A_{tot}(0) - A_{tot}(t)}{P_{0,tot}\ t}$
Time $(t_0 - t)$ / s

Fig. SI-S1-13:

SI: S2

Photokinetics of $XY_1(2\Phi, 1k)$ reaction

Reaction:

$XY_1(2\Phi, 1k)$ **UV** only

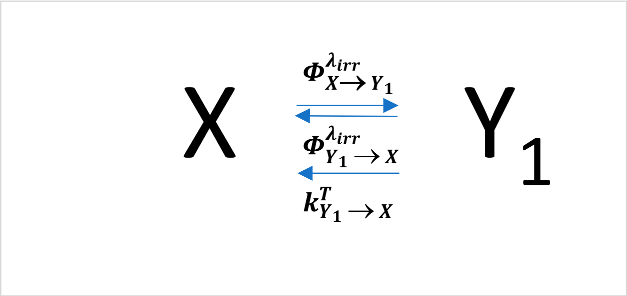

reaction parameters:

$C_X^{Lp,\Delta\lambda,T}(0)$ / M	l_{irr} / cm	$k^T_{Y_1 \rightarrow X}$ / s^{-1}	$k^T_{Y_2 \rightarrow Y_1}$ / s^{-1}	h / s	λ_{obs} /nm	l_{obs} / cm
4 10^{-5}	0.4	0.0028	-	10	283	0.4

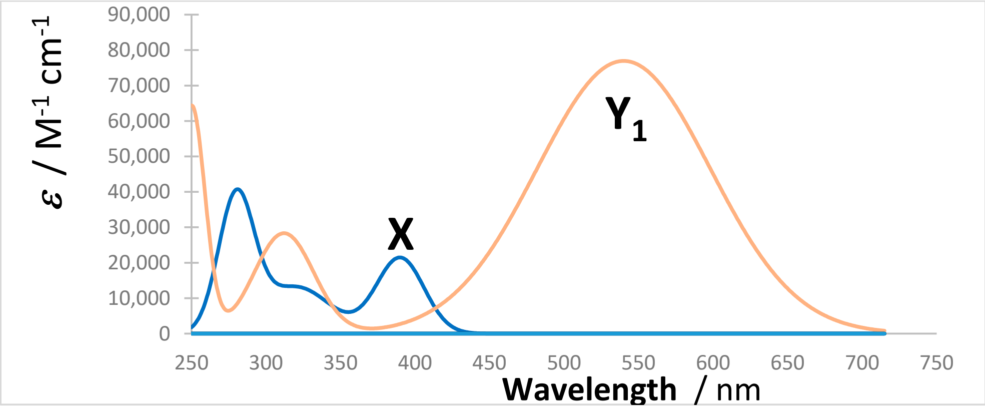

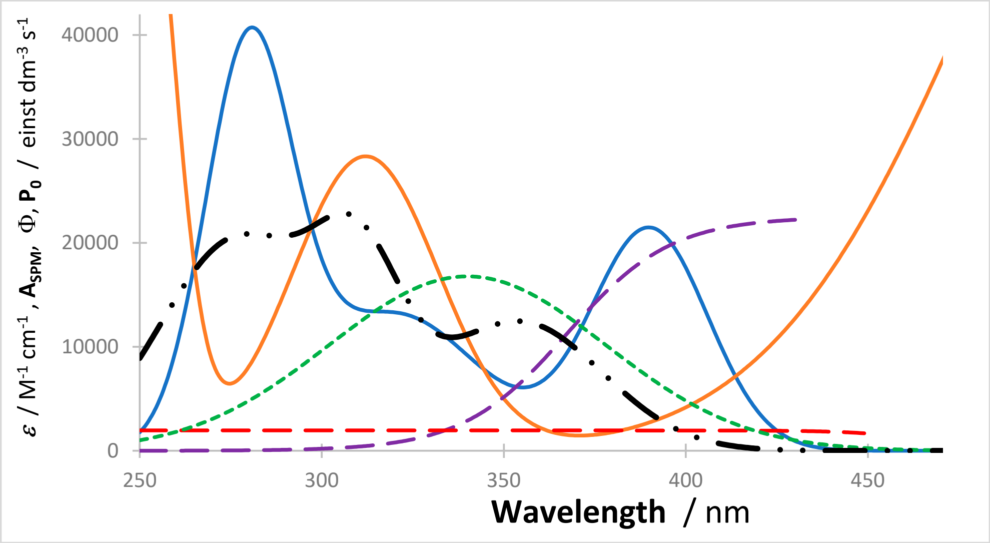

Blue —: $\varepsilon_X^{\lambda_{irr}}$; Orange —: $\varepsilon_{Y_1}^{\lambda_{irr}}$; Green ----: $P_0^{\lambda_{irr}} \times 10^{11}$; Black — · · —: $A_{SPM}^{\lambda_{irr}} \times 10^4$;

Purple — —: $\Phi^{\lambda_{irr}}_{X \rightarrow Y_1} \times 3\ 10^5$; Red — —: $\Phi^{\lambda_{irr}}_{Y_1 \rightarrow X} \times 3\ 10^5$

Fitting equation:

$$C(t) = ww + w_1 \log(1 + cc\, e^{-k_1 t}) + s_1\, e^{-k_{s1} t}$$

Fitting data:

CX: SSE: 10^{-16} TO 10^{-22} ; RMSE: 10^{-10} TO 10^{-12} ;
Atot: SSE: 10^{-6} TO 10^{-10} ; RMSE: 10^{-5} TO 10^{-7} ;
r^2: 1

Initial rates:

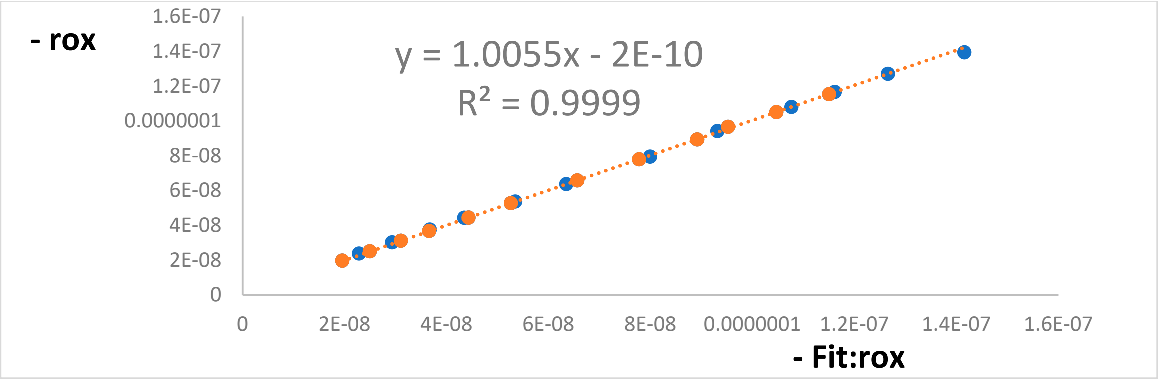

SPM Effect:
Table S2-SPM.

	a	b	c	d	e	f	g	h	i	j	k	L
A(SPM) @308nm	0	.315	.631	.946	1.58	2.52	4.1	5.68	7.89	10.25	19.95	14.19

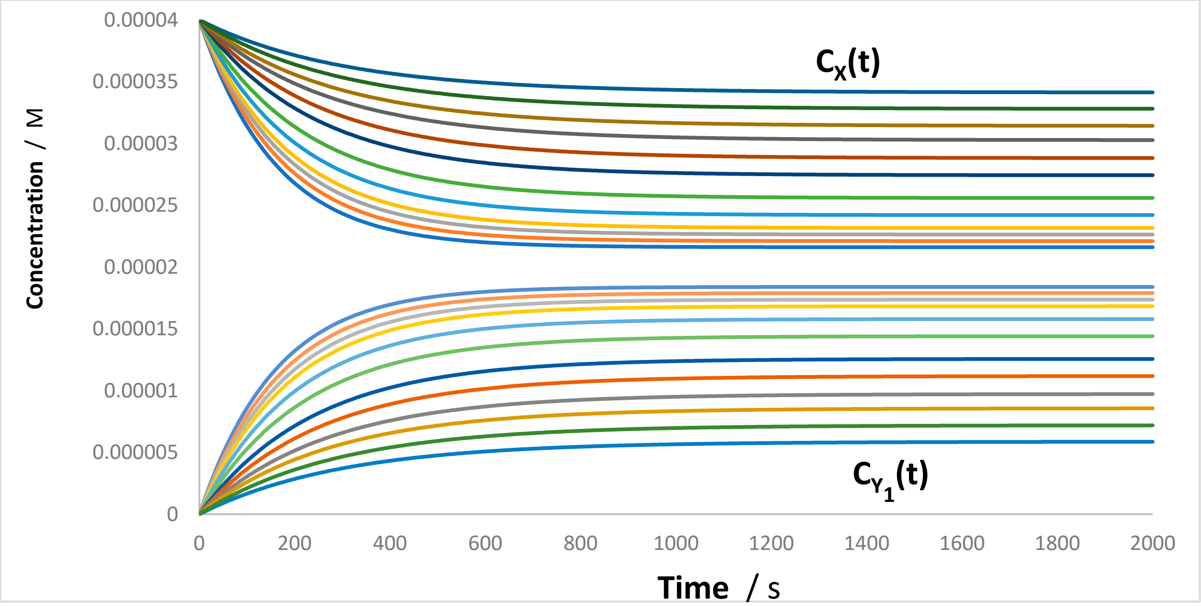

Total absorbance = *f*(**A(SPM)**)

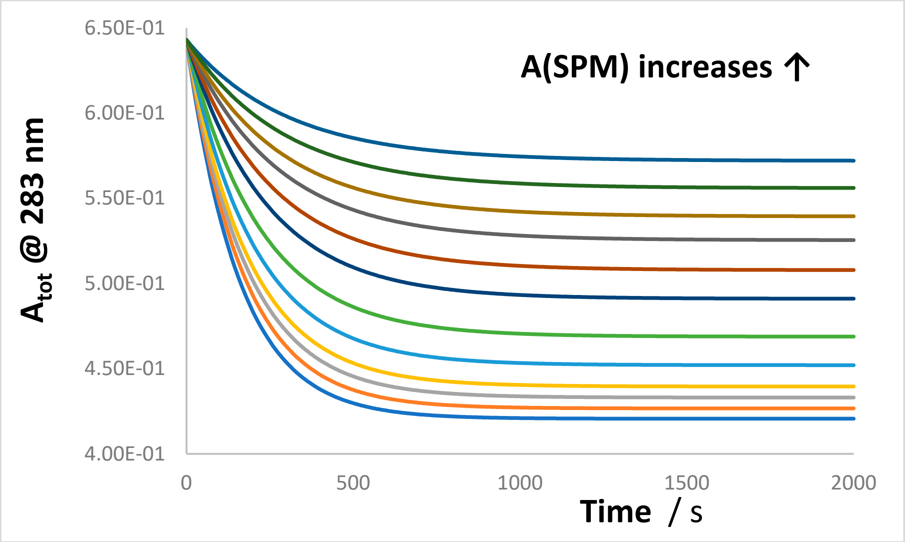

$-r_{oX} = f(\mathbf{A_{tot}(\infty)}$ **@ 283 nm**)

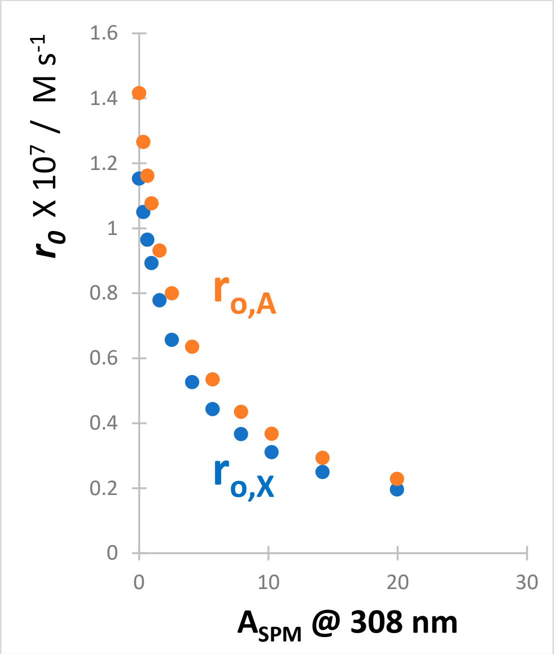

PY:

$$PY_X^{Lp,\Delta\lambda,T} = -\frac{r_{0,X}^{Lp,\Delta\lambda,T}}{P_0^{Lp,\Delta\lambda}} \qquad or \qquad PY_A^{Lp,\Delta\lambda,T} = -\frac{r_{0,A}^{Lp,\Delta\lambda,T}}{P_0^{Lp,\Delta\lambda}} \qquad (\mathbf{S2-1})$$

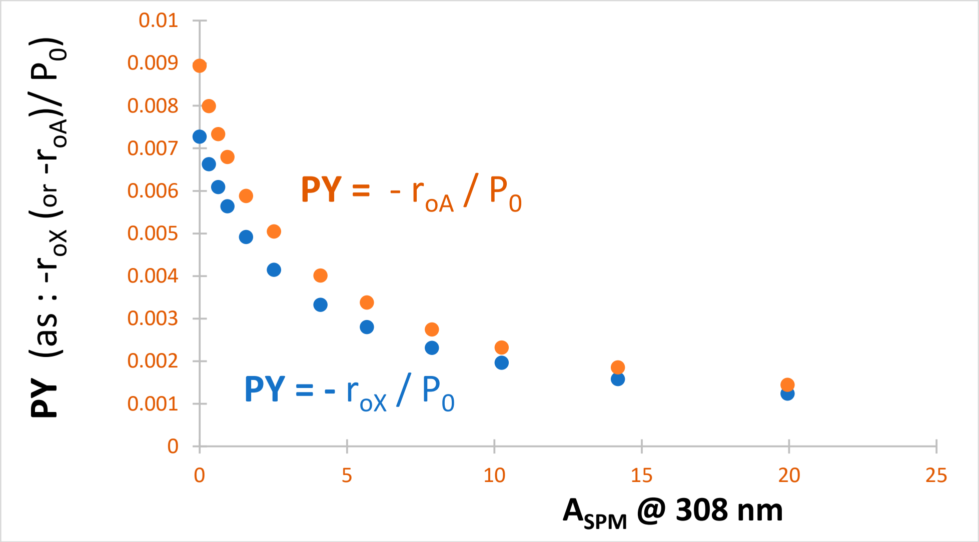

PY:

$$PY_{Y_j}^{Lp,\Delta\lambda,T} = \frac{C_X^{Lp,\Delta\lambda,T}(0) - C_X^{Lp,\Delta\lambda,T}(t)}{P_0^{Lp,\Delta\lambda}\, t} \qquad (S2-2)$$

PY is calculated for each trace of X at a different SPM concentration as given in Table SPM.

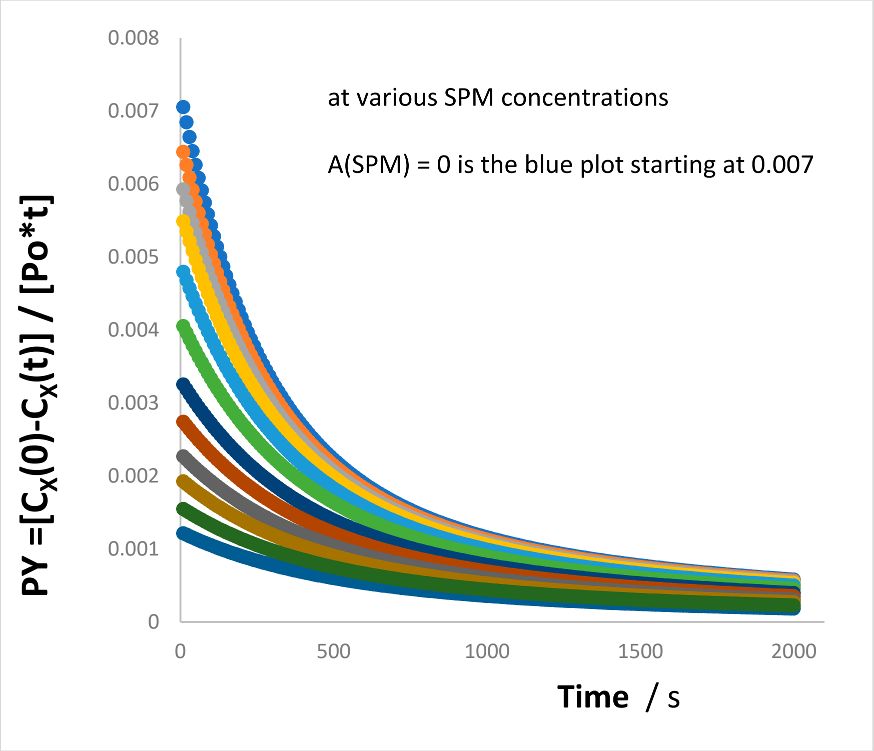

SI: S3

Photokinetics of $XY_3(6\Phi, 5k)$ reaction

Reaction:

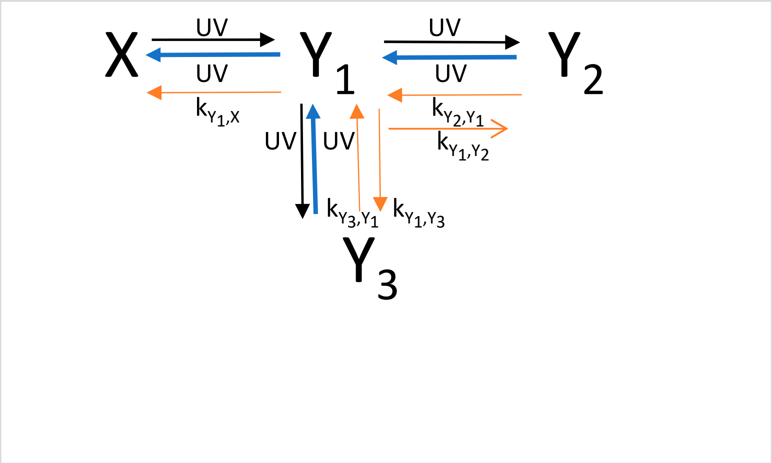

reaction parameters:

$C_X^{Lp,\Delta\lambda,T}(\mathbf{0})$ / M	l_{irr} / cm	h / s	λ_{obs} /nm	l_{obs} / cm		
3.58 10^{-5}	0.73	2	356	0.73		
$k^T_{Y_1\to X}$ / s^{-1}	$k^T_{Y_1\to Y_2}$ / s^{-1}	$k^T_{Y_2\to Y_1}$ / s^{-1}	$k^T_{Y_1\to Y_3}$ / s^{-1}	$k^T_{Y_3\to Y_1}$ / s^{-1}		
0.008	0.0047	0.00182	0.00255	0.0033		

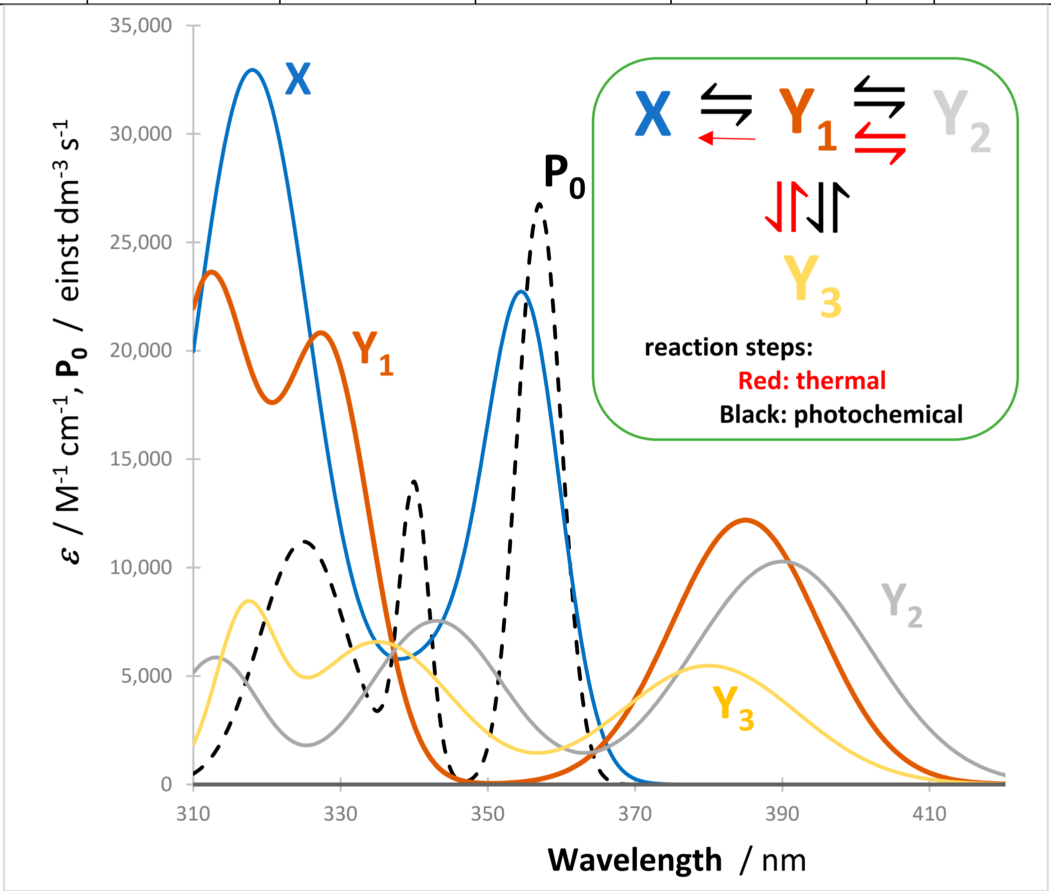

Φ=f(λ)

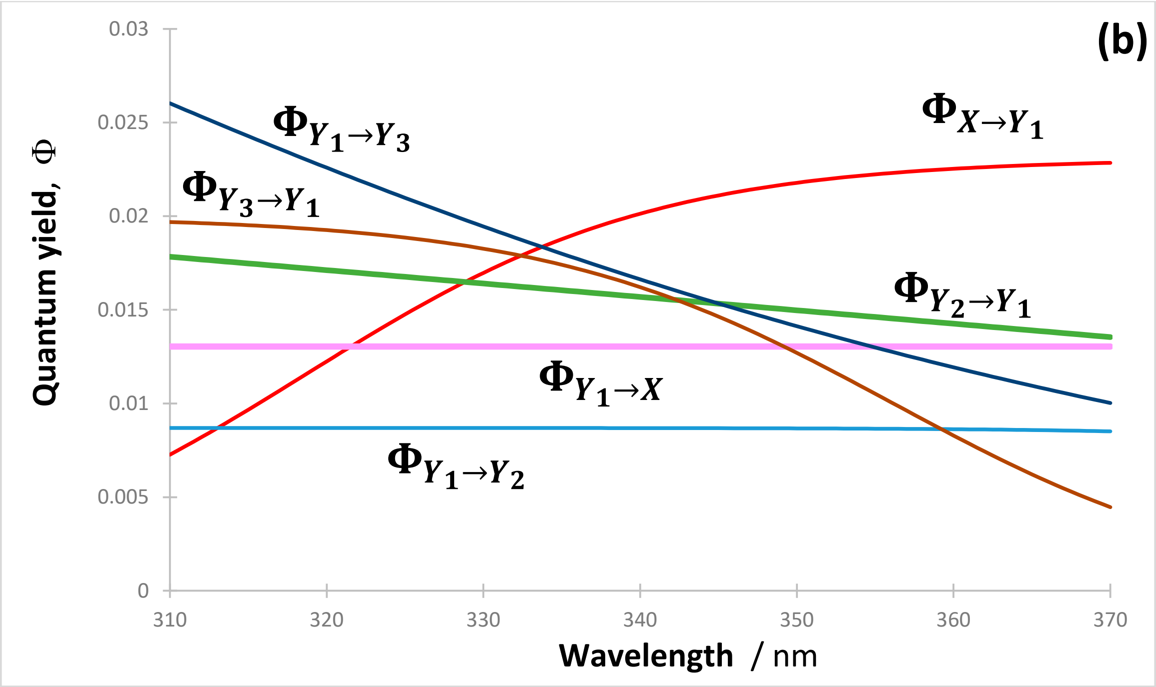

Fitting equation:

$$C(t) = ww + w_1 \log(1 + cc\, e^{-k_1 t}) + w_2 \log(1 + cc\, e^{-k_2 t}) + s_1\, e^{-k_{s1} t}$$

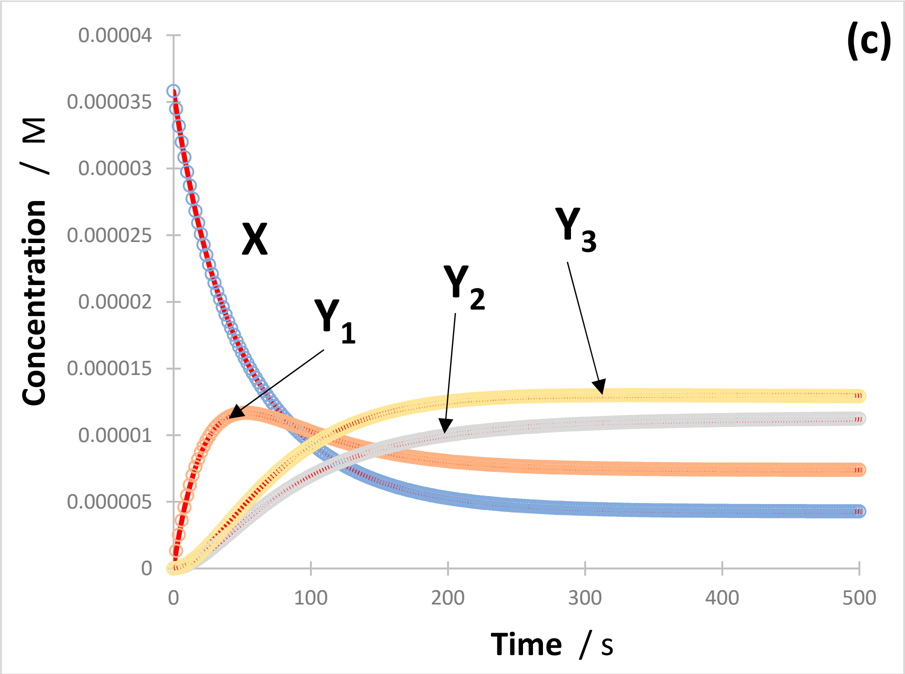

EFFECT of Po: concentration traces

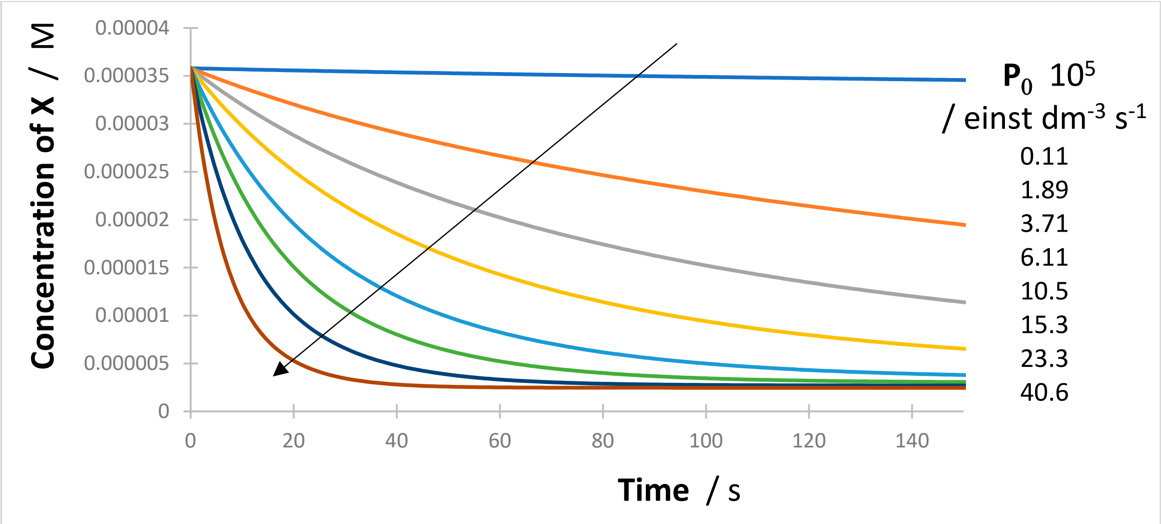

EFFECT of Po: total absorbance traces

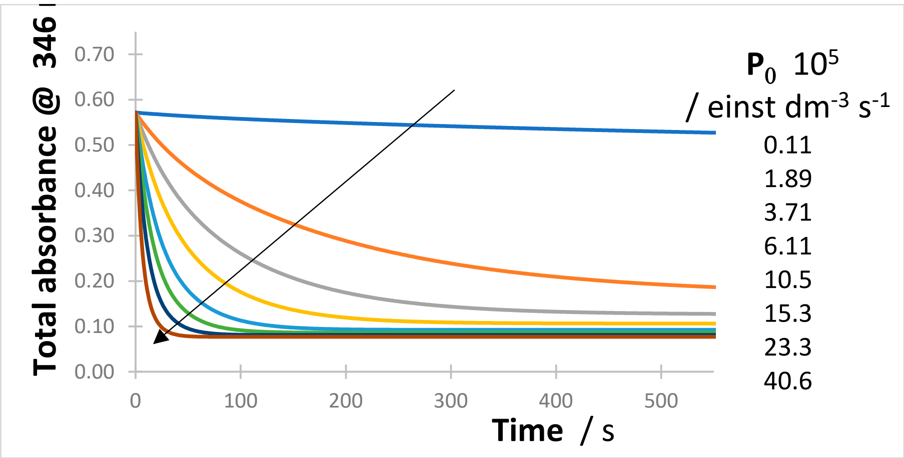

EFFECT of Po: on r_0

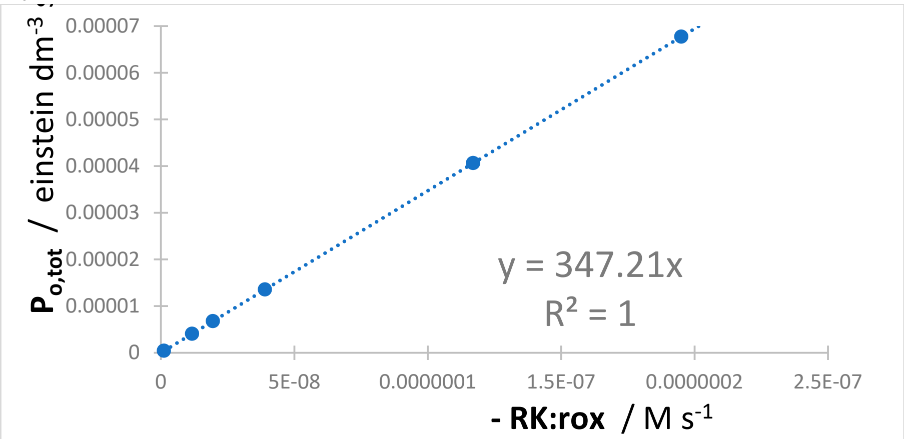

EFFECT of Po: on PY (concentration data)

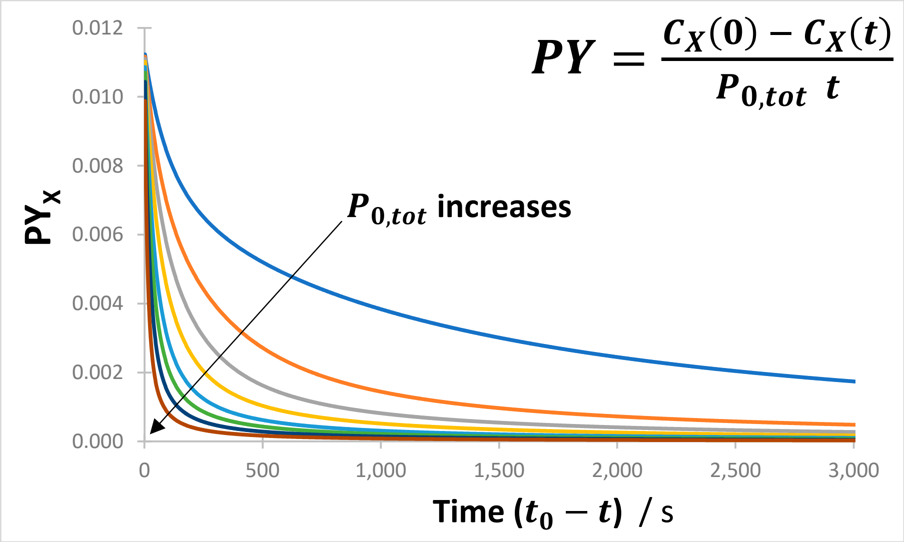

EFFECT of Po: on PY (absorbance data)

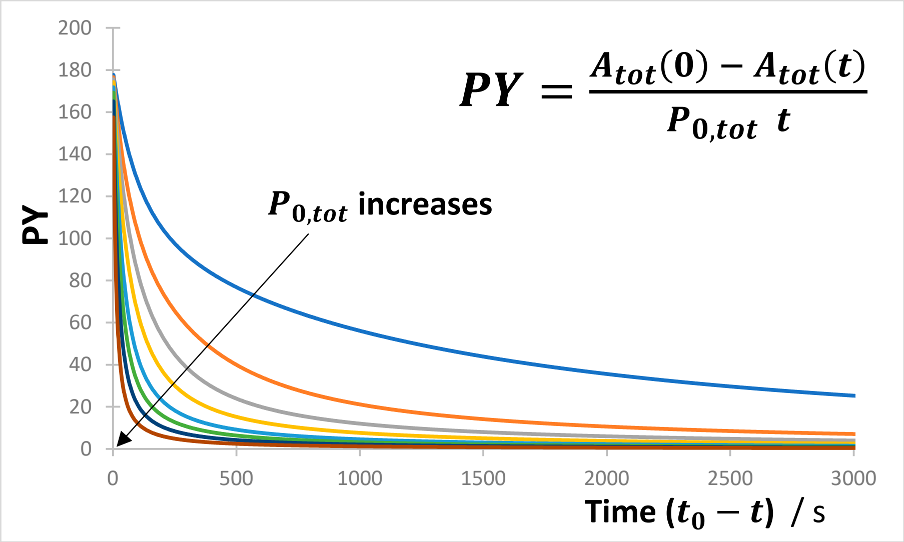

EFFECT of T: on PY (concentration data of Y3)

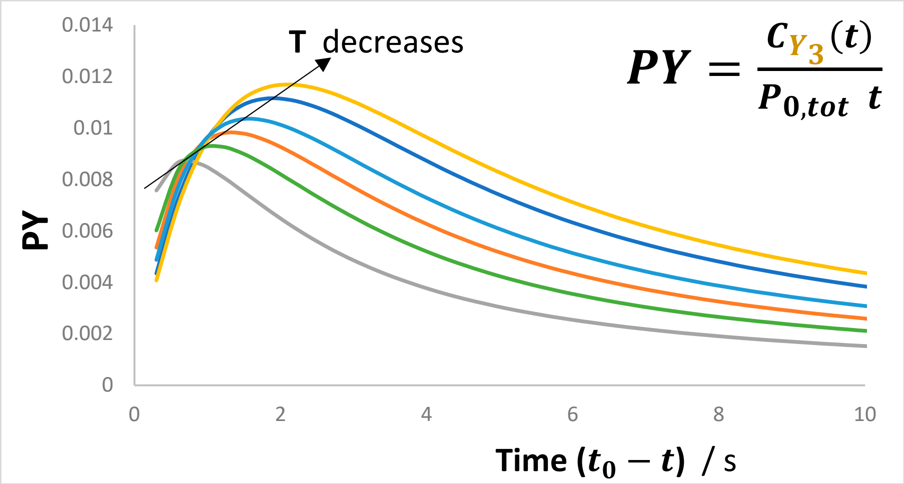

EFFECT of T:

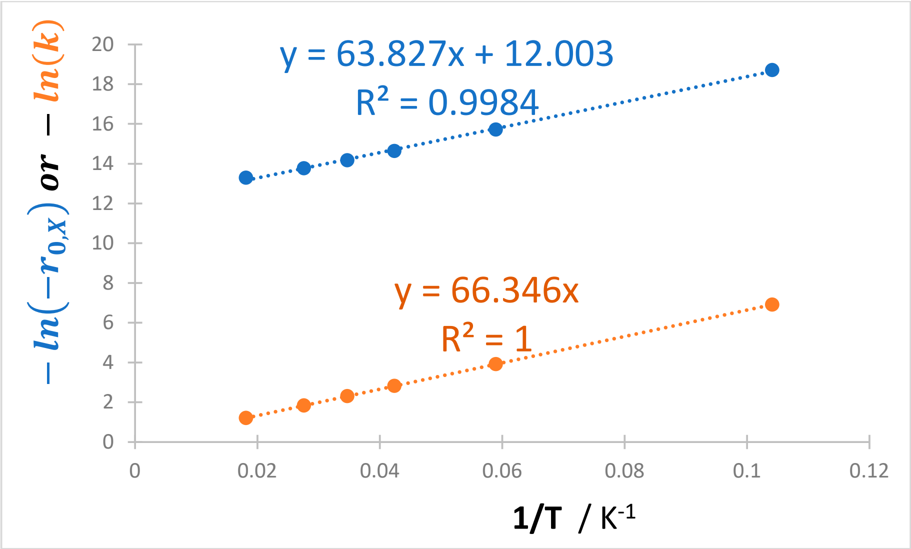

y = 63.827x + 12.003
R² = 0.9984
y = 66.346x
R² = 1
− ln(−r0,X) or − ln(k)
1/T / K-1
20
18
16
14
12
10
8
6
4
2
0
0
0.02
0.04
0.06
0.08
0.1
0.12

EFFECT of T:

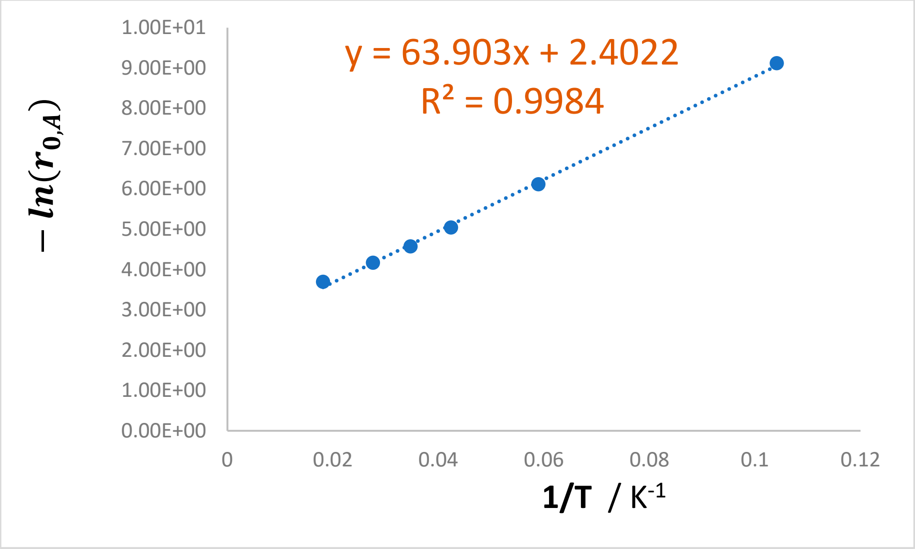

y = 63.903x + 2.4022
R² = 0.9984
− ln(r0,A)
1/T / K-1
1.00E+01
9.00E+00
8.00E+00
7.00E+00
6.00E+00
5.00E+00
4.00E+00
3.00E+00
2.00E+00
1.00E+00
0.00E+00
0
0.02
0.04
0.06
0.08
0.1
0.12

Kinetic elucidation of photothermal kinetics
An illustration example for $XY_2(3\Phi,2k)$ mechanism

The example selected here is photothermal reaction $XY_2(3\Phi,2k)$. The mechanism of the reaction is the one proposed in the literature for the photokinetics of naphthopyrans (Frigoli *et al.*, 2015; Gierczyk *et al.*, 2022).

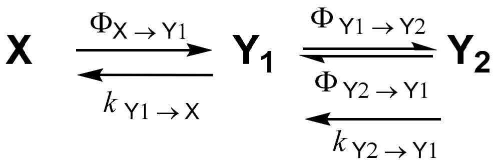

Scheme **S4-1**

The RK-calculation was fed by the reaction parameters given in Table S4-1.

Table **S4-1**

$C_X^{\lambda_{irr},T}(0)$ / M	l_{irr} / cm	l_{obs} / cm	h	$P_{0,tot}^{\lambda_{irr}}$ / M^{-1} s^{-1}	λ_{irr} /nm	λ_{obs} /nm	
1.33 10^{-5}	2	2	3	3.13 10^{-6}	341	341	
$k_{Y_1\to X}^T$ / s^{-1}	$k_{Y_2\to Y_1}^T$ / s^{-1}	$\Phi_{X\to Y_1}^{\lambda_{irr}}$	$\Phi_{Y_1\to Y_2}^{\lambda_{irr}}$	$\Phi_{Y_2\to Y_1}^{\lambda_{irr}}$	$\varepsilon_X^{\lambda_{irr}}$ / M^{-1} cm^{-1}	$\varepsilon_{Y_1}^{\lambda_{irr}}$ / M^{-1} cm^{-1}	$\varepsilon_{Y_2}^{\lambda_{irr}}$ / M^{-1} cm^{-1}
0.015	0.007	2.895 10^{-2}	3.039 10^{-2}	2.885 10^{-2}	8.8792 10^3	8.6513 10^3	1.3770 10^3

The elucidation journey:

1- *The knowns*

1a. The reaction is performed under monochromatic light (at $\lambda_{irr} = 341\ nm$).

1b. It is necessary that the number of species involved in the mechanism is known. For the case studied here there are three species: $\boldsymbol{X = Y_0}$, $\boldsymbol{Y_1}$ and $\boldsymbol{Y_2}$.

1c. The mechanism of the reaction is supposed known as Scheme **S4-1**.

1d. The reactant is thermally stable and its extrinsic parameters are known, as given in Table 5-1.

1c. The traces of the individual species concentrations as well as the total absorbance of the medium are available

2- *The unknowns*

2a. The unknowns of the kinetics to be determined by the elucidation method are seven: 3 quantum yield, 2 absorption coefficients, and 2 thermal rate-constants. Their values in Table S5-1 are ignored (supposed unknown) and will serve as references to the ones obtained by the elucidation method (*vide infra*).

3- *Fitting equations of the traces*

3a. The fitting equations for the photothermal reaction traces, that include two Φ-order and two 1st-ordre terms. The applied equations for the fitting of the present data correspond to the general equations Eqs.6 and 9. Here the number of Φ-order terms in the photothermal kinetic equations ($i_{\Phi,j} = i_{\Phi,j,A} = 2$) is less than the photochemical reaction-steps ($n_{\Phi,j} = 3$), these numbers are equal for the thermal reaction ($n_{\Delta,j} = i_{\Delta,j} = i_{\Delta,j,A} = 2$). Observe that the equations share the following coefficients cc^{Φ}, k_{1j}^{Φ}, k_{2j}^{Φ}, k_{1j}^{Δ}, and k_{2j}^{Δ}. Such communality of coefficients in the equations is not a requirement, but is shown here to indicate the coherence of the kinetic treatment.

$$C_{Y_j}^{\lambda_{irr},T}(t) = \omega_j^0 + \omega_{1j}^{\Phi}\, Log\left(1 + cc^{\Phi} e^{-k_{1j}^{\Phi}\,t}\right) + \omega_{2j}^{\Phi}\, Log\left(1 + cc^{\Phi} e^{-k_{2j}^{\Phi}\,t}\right) + \omega_{1j}^{\Delta}\, e^{-k_{1j}^{\Delta}\,t} + \omega_{2j}^{\Delta}\, e^{-k_{2j}^{\Delta}\,t} \qquad (\boldsymbol{S4-1})$$

$$A_{tot}^{\lambda_{irr},T/\lambda_{obs}}(t) = A_{tot}^{\lambda_{irr},T/\lambda_{obs}}(\infty) + \omega_{1,A}^{\Phi}\, Log\left(1 + cc^{\Phi} e^{-k_{1j}^{\Phi}\,t}\right) + \omega_{2,A}^{\Phi}\, Log\left(1 + cc^{\Phi} e^{-k_{2j}^{\Phi}\,t}\right) + \omega_{1,A}^{\Delta}\, e^{-k_{1j}^{\Delta}\,t} + \omega_{2,A}^{\Delta}\, e^{-k_{2j}^{\Delta}\,t} \qquad (\boldsymbol{S4-2})$$

3b. The fitting equation for the thermal reaction traces, are

$$C_{Y_j}^{T}(t) = \omega_j^{0,\Delta'} + \omega_{1j}^{\Delta'}\, e^{-\,k_{1j}^{\Delta}\,t} + \omega_{2j}^{\Delta'}\, e^{-\,k_{2j}^{\Delta}\,t} \qquad (\boldsymbol{S4-3})$$

$$A_{tot}^{T/\lambda_{obs}}(t) = A_{tot}^{T/\lambda_{obs}}(\infty) + \omega_{i,A}^{\Delta'}\, e^{-\,k_{1j}^{\Delta}\,t} + \omega_{i,A}^{\Delta'}\, e^{-\,k_{2j}^{\Delta}\,t} \qquad (\boldsymbol{S4-4})$$

3c. The fitting parameters of the traces are given in Table **S4-2**.

Table **S4-2**

	Photothermal reaction			
	$C_X^{Lp,\Delta\lambda,T}(t)$	$C_{Y_1}^{Lp,\Delta\lambda,T}(t)$	$C_{Y_2}^{Lp,\Delta\lambda,T}(t)$	$A_{tot}^{Lp,\Delta\lambda,T/\lambda_{obs}}(t)$
cc^{Φ}	1.1	1.1	1.1	1.1
k_{1j}^{Φ}	0.01173	0.01173	0.01173	0.01173
k_{2j}^{Φ}	0.006935	0.006935	0.006935	0.006935
ω_{1j}^{Φ} or $\omega_{1,A}^{\Phi}$	5.38 10^{-5}	-6.23 10^{-5}	8.48 10^{-6}	1.29 10^{-1}
ω_{2j}^{Φ} or $\omega_{2,A}^{\Phi}$	1.62 10^{-4}	-1.95 10^{-4}	3.28 10^{-5}	4.62 10^{-1}
k_{1j}^{Δ}	0.015	0.015	0.015	0.015
k_{2j}^{Δ}	0.007	0.007	0.007	0.007
ω_{1j}^{Δ} or $\omega_{1,A}^{\Delta}$	1.36 10^{-5}	-1.64 10^{-5}	2.79 10^{-6}	3.86 10^{-2}
ω_{2j}^{Δ} or $\omega_{2,A}^{\Delta}$	-8.07 10^{-5}	9.72 10^{-5}	-1.66 10^{-5}	-2.33 10^{-1}
ω_j^{0} or $A_{tot}^{Lp,\Delta\lambda,T/\lambda_{obs}}(\infty)$	1.07 10^{-5}	2.04 10^{-6}	5.20 10^{-7}	2.40 10^{-1}
SSE	6.52 10^{-15}	6.48 10^{-15}	1.34 10^{-13}	2.45 10^{-9}
r^2	0.999	0.998	0.981	0.999
$RMSE$	3.63 10^{-9}	3.61 10^{-9}	1.65 10^{-8}	2.22 10^{-6}

4- *Determination of the absorption coefficients at λ_{irr}*

4a. we have considered here that $\lambda_{irr} = \lambda_{obs} = 2cm$.

4b. ε_X : since the absorption spectrum and the initial concentration of X are known, the absorptivity is worked out from the Beer-Lambert law at any wavelength (including λ_{irr}).

$$\varepsilon_X = \frac{A_{tot}^{Lp,\Delta\lambda,T/\lambda_{obs}}(t)}{C_X^{Lp,\Delta\lambda,T}(0)\; l_{irr}} \qquad (\boldsymbol{S4-5})$$

4c. ε_{Y_1} and ε_{Y_2}: to solve for these two absorptivities, a system of two linear and linearly-independent equations is required. These are derived from the Beer-Lambert equation of the total absorbance of the photochemical reaction at two different time intervals, e.g., at 129 and 348 s (Eqs.S4-6 and S4-7). The right hand-side terms of the equations are defined by available parameters. The values of concentrations and absorbancies occurring in those equations, at the given times, are worked out from the appropriate Eqs.S4-1 and S4-2. Solving a system of linear equation can be achieved by many software including Microsoft Excel.

$$\varepsilon_{Y_1}\, C_{Y_1}^{Lp,\Delta\lambda,T}(129) + \varepsilon_{Y_2}\, C_{Y_2}^{Lp,\Delta\lambda,T}(129) = \frac{A_{tot}^{Lp,\Delta\lambda,T/\lambda_{obs}}(129) - \varepsilon_X\, C_X^{Lp,\Delta\lambda,T}(129)\; l_{irr}}{l_{irr}} \qquad (\boldsymbol{S4-6})$$

$$\varepsilon_{Y_1}\, C_{Y_1}^{Lp,\Delta\lambda,T}(348) + \varepsilon_{Y_2}\, C_{Y_2}^{Lp,\Delta\lambda,T}(348) = \frac{A_{tot}^{Lp,\Delta\lambda,T/\lambda_{obs}}(348) - \varepsilon_X\, C_X^{Lp,\Delta\lambda,T}(348)\; l_{irr}}{l_{irr}} \qquad (\boldsymbol{S4-7})$$

4d. the elucidation results for the absorptivities are compared, in Table **S4-3**, to the data that originally fed the RK calculation. The % errors found are less than 5%.

Table **S4-3**

	Absorption coefficients of the species at λ_{irr}		
	RK fed	Elucidation method	% Err
ε_X	8879.20	8879.20	0
ε_{Y_1}	8651.31	8491.644	1.85
ε_{Y_2}	13770.67	14298.52	3.83

5- *Determination of the species quantum yield at λ_{irr}*

5a. $\Phi_{X\to Y_1}$: because the thermally stable reactant only reacts photochemically to yield Y_1, this reaction quantum yield (Eq.S5-8) can be worked out from the theoretical equation of the initial-rate (Eq.5).

$$\Phi_{X\to Y_j}^{\lambda_{irr}} = -\frac{Theor{:}\,r_{0,X}^{Lp,\Delta\lambda,T}}{P_0^{\lambda_{irr}}\left(1-10^{-A_{tot}^{\lambda_{irr}}(0)}\right)} \qquad (\boldsymbol{S4-8})$$

The values of $P_0^{\lambda_{irr}}$ and $A_{tot}^{\lambda_{irr}}(0)$ are obtained from Tables **S4-1** and **S4-3**, and the numerical value of $r_{0,X}^{Lp,\Delta\lambda,T}$ is worked out from the fitting equation (Eq.8) as Eq.S4-9, with the parameters of the latter provided by Table **S4-2**.

$$Fit{:}\,r_{0,X}^{Lp,\Delta\lambda,T} = -\frac{1}{ln(10)}\frac{\omega_{10}^{\Phi}\,cc^{\Phi}\,k_{10}^{\Phi}+\omega_{11}^{\Phi}\,cc^{\Phi}\,k_{11}^{\Phi}}{1+cc^{\Phi}} - \omega_{10}^{\Delta}\;k_{10}^{\Delta} - \omega_{11}^{\Delta}\;k_{11}^{\Delta} \qquad (\boldsymbol{S4-9})$$

the % error between the value obtained from the RK calculation ($RK{:}\,r_{0,X}^{Lp,\Delta\lambda,T}$) and that from Eq.S4-9 ($Fit{:}\,r_{0,X}^{Lp,\Delta\lambda,T}$) is relatively small (Table **S4-4**).

Table **S4-4**

	Initial reactant-rate		
	$RK{:}\,r_{0,X}^{Lp,\Delta\lambda,T}$	$Fit{:}\,r_{0,X}^{Lp,\Delta\lambda,T}$	% Err
$r_{0,X}^{Lp,\Delta\lambda,T}$	- 3.80146 10^{-8}	- 3.8675 10^{-8}	1.74

5b. The determination of the remaining two quantum yields $\Phi_{Y_1\to Y_2}$ and $\Phi_{Y_2\to Y_1}$ requires solving a system of two equations. These might be provided by the rate equations of the photothermal reactions (Eq.4) of either species. For simplicity, in the particular case of the reaction investigated here, the equation relative to Y_2 is used for two time intervals (129 and 348 s).

$$r_{0,Y_2}^{Lp,\Delta\lambda,T}(t) = -\,\Phi_{Y_2\to Y_1}^{\lambda_{irr}}\,P_{aY_2}^{\lambda_{irr}}(t) + \Phi_{Y_1\to Y_2}^{\lambda_{irr}}\,P_{aY_1}^{\lambda_{irr}}(t) - k_{Y_2\to Y_1}^{\Delta}\,C_{Y_2}^{Lp,\Delta\lambda,T}(t) \qquad (\boldsymbol{S4-10})$$

which gives

$$-\,\Phi_{Y_2\to Y_1}^{\lambda_{irr}}\,P_{aY_2}^{\lambda_{irr}}(129) + \Phi_{Y_1\to Y_2}^{\lambda_{irr}}\,P_{aY_1}^{\lambda_{irr}}(129) = r_{0,Y_2}^{Lp,\Delta\lambda,T}(129) + k_{Y_2\to Y_1}^{\Delta}\,C_{Y_2}^{Lp,\Delta\lambda,T}(129) \qquad (\boldsymbol{S4-11})$$

$$-\,\Phi_{Y_2\to Y_1}^{\lambda_{irr}}\,P_{aY_2}^{\lambda_{irr}}(348) + \Phi_{Y_1\to Y_2}^{\lambda_{irr}}\,P_{aY_1}^{\lambda_{irr}}(348) = r_{0,Y_2}^{Lp,\Delta\lambda,T}(348) + k_{Y_2\to Y_1}^{\Delta}\,C_{Y_2}^{Lp,\Delta\lambda,T}(348) \qquad (\boldsymbol{S4-12})$$

The numerical values of the right hand-side terms of the above equations are accessible from Table S4-2 ($k_{Y_2\to Y_1}^{\Delta}$), Eq.S4-1 for Y2 and its parameters' values from Table **S4-2** ($C_{Y_2}^{Lp,\Delta\lambda,T}(129)$ and $C_{Y_2}^{Lp,\Delta\lambda,T}(348)$), and Eq.7 and Table **S4-2** for the calculation of $r_{0,Y_2}^{Lp,\Delta\lambda,T}(129)$ and $r_{0,Y_2}^{Lp,\Delta\lambda,T}(248)$ values. The numerical values of the coefficients of the quantum yields ($P_{aY_1}^{\lambda_{irr}}$ and $P_{aY_2}^{\lambda_{irr}}$, respectively, Eqs.S4-13 and 14), in the left hand-side of Eqs.S4-11 and S4-12, are calculated from Eq.2 for the indicated time intervals. The values of $A_{Y_1}^{\lambda_{irr}}(t)$ and $A_{Y_2}^{\lambda_{irr}}(t)$ are worked out from the corresponding Beer-Lambert law, using the concentration of the species at the given time interval (obtained from the trace, Eqs.S4-1) and the corresponding absorption coefficient (Table **S4-3**). The values of $A_{tot}^{\lambda_{irr}}(t)$ is calculated from Eq.S4-2.

$$P_{a_{Y_1}}^{\lambda_{irr}}(t) = \frac{A_{Y_1}^{\lambda_{irr}}(t)}{A_{Y_1}^{\lambda_{irr}}(t) + A_{Y_2}^{\lambda_{irr}}(t)} P_0^{\lambda_{irr}} \left(1 - 10^{- - A_{Y_1}^{\lambda_{irr}}(t) - A_{Y_2}^{\lambda_{irr}}(t)}\right) \quad (\boldsymbol{S4-13})$$

$$P_{a_{Y_2}}^{\lambda_{irr}}(t) = \frac{A_{Y_2}^{\lambda_{irr}}(t)}{A_{Y_1}^{\lambda_{irr}}(t) + A_{Y_2}^{\lambda_{irr}}(t)} P_0^{\lambda_{irr}} \left(1 - 10^{- A_{Y_1}^{\lambda_{irr}}(t) - A_{Y_2}^{\lambda_{irr}}(t)}\right) \quad (\boldsymbol{S4-13})$$

The solution of the system of equation can be delivered by Microsoft Excel, as presented in Table **S4-5**.

Table **S4-5**

	Quantum yields of the species at λ_{irr}		
	RK fed	Elucidation method	% Err
$\Phi_{X \rightarrow Y_1}^{\lambda_{irr}}$	0.02895	0.02945	1.73
$\Phi_{Y_1 \rightarrow Y_2}^{\lambda_{irr}}$	0.03039	0.03094	1.78
$\Phi_{Y_2 \rightarrow Y_1}^{\lambda_{irr}}$	0.02885	0.02765	4.15

The elucidation procedure presented here for $XY_2(3\Phi, 2k)$ can be generalised and applied to elucidate the kinetics of any system (where the number of linear equations to be solved might be different).
